# Constructing Co_3_O_4_/g-C_3_N_4_ Ultra-Thin Nanosheets with Z-Scheme Charge Transfer Pathway for Efficient Photocatalytic Water Splitting

**DOI:** 10.3390/nano11123341

**Published:** 2021-12-09

**Authors:** Yuan Guo, Wanqing Liu, Wei Duan, Siyu Wang, Liqun Jia, Guoqing Zhang, Baolin Zhu, Weiping Huang, Shoumin Zhang

**Affiliations:** Department of Chemistry, Key Laboratory of Advanced Energy Material Chemistry (MOE), TKL of Metal and Molecule Based Material Chemistry, Nankai University, Tianjin 300071, China; 2120190685@mail.nankai.edu.cn (Y.G.); 2120200715@mail.nankai.edu.cn (W.L.); dduan0417@163.com (W.D.); siyu2142021@163.com (S.W.); jlq15064153315@163.com (L.J.); zhangguoqing9401@163.com (G.Z.); zhubaolin@nankai.edu.cn (B.Z.); hwp914@nankai.edu.cn (W.H.)

**Keywords:** Co_3_O_4_/g-C_3_N_4_ ultra-thin nanosheets, Z-scheme charge transfer pathway, photocatalytic, water splitting, H_2_ evolution

## Abstract

Photocatalytic water splitting for hydrogen generation is a significant pathway for sustainable energy conversion and production. The photocatalysts with a Z-scheme water splitting charge transfer pathway is superior due to the good separation and migration ability of photoexcited charge carriers. Herein, Co_3_O_4_/g-C_3_N_4_ photocatalysts with Z-scheme charge transfer pathway were successfully constructed by an electrostatic interaction-annealing method. The as-prepared Co_3_O_4_/g-C_3_N_4_ ultra-thin nanosheets were tested and analyzed by XRD, EA, ICP, SEM, TEM, AFM, XPS, UV-Vis DRS, PL and photoelectrochemical measurements. Moreover, the influences of fabrication parameters on performance of Co_3_O_4_/g-C_3_N_4_ catalysts were investigated, and 0.5% Co_3_O_4_/g-C_3_N_4_ exhibited the optimal activity. Based on the characterization and catalytic performance, the Z-scheme charge transfer pathway of Co_3_O_4_/g-C_3_N_4_ was established and put forward. To further improve the catalytic performance of Co_3_O_4_/g-C_3_N_4_, 0.5% Pt was added as a co-catalyst. The obtained Pt/0.5% Co_3_O_4_/g-C_3_N_4_ was recyclable and remained the original catalytic water splitting performance within 20 h. The modification of Co_3_O_4_ and Pt improved the separation and migration of e^−^ and h^+^, and induced the increased hydrogen evolution rate of g-C_3_N_4_.

## 1. Introduction

Photocatalytic hydrogen generation is a high efficiency, environmentally friendly and economically practical technology for utilizing solar energy [1,2,3]. It promises a sustainable alternative via semiconductors to address environmental issues and energy shortages all around the world [4,5,6]. The strong optical absorption ability, high separation and migration efficiency and stability are major factors for the photocatalysts’ activity for hydrogen evolution [7,8]. Now efforts have been devoted to fabricating more and more effective semiconductors for solar-energy utilization and conversion, majorly categorized as metal oxides, amorphous (oxy)-hydroxides, (oxy)nitrides, (oxy)sulphides and polymeric catalysts [9,10].

Graphitic carbon nitride (g-C_3_N_4_) is a robust and nontoxicity polymeric catalyst with good properties [11]. It was constructed by the polymerization method as a photocatalytic water splitting catalyst in 2009 [12]. Up to now, g-C_3_N_4_ has been widely developed as an emerging and prospective catalyst in different areas, for example photocatalytic degradation of pollution [13], hydrogen evolution through photocatalytic water splitting [14] and CO_2_ reduction [15]. Nowadays g-C_3_N_4_ with various morphologies have emerged, such as quantum dots [16,17], nanotubes [18,19], bulk g-C_3_N_4_ and g-C_3_N_4_ nanosheets [20]. Two-dimensional g-C_3_N_4_ nanosheets have been intensively explored because of their outstanding photocatalytic performance in the past few years [20]. For example, the hydrogen generation ability of two-dimensional g-C_3_N_4_ nanosheets was improved by Lu and co-workers [21]. It is reported that g-C_3_N_4_ nanosheets have an improved catalytic performance compared with bulk g-C_3_N_4_, benefiting from its enlarged redox potentials [22], and prolonged charge carrier lifetime. As has been reported, a short transfer path can be obtained in the g-C_3_N_4_ nanosheets [22]. Therefore, efficient performance of g-C_3_N_4_ with 2D ultra-thin structure can be anticipated. In 2015, Liu and co-workers reported that the as-synthesized g-C_3_N_4_ nanosheets possessed an efficient water splitting ability by nature-inspired environment “phosphorylation” [23]. Unfortunately, g-C_3_N_4_ has a lot of defects, such as an inner self-combination of electrons and holes, limited light response ability, and so on [24]. To overcome the above shortcomings and greatly enhance its photocatalytic H_2_ production activity, semiconductor composite [25], dye sensitization, and co-catalyst modification [26] have been tried.

Among the semiconductor composites, the Z-scheme charge transfer pathway is a practical strategy. Its prominent advantages are the efficient separation of e^−^ and h^+^ at conduction band edges (E_CB_) of one semiconductor and valence band edges (E_VB_) of other semiconductors, respectively. Therefore, it can inhibit the inner self-combination of electrons and holes. The transfer process schematic diagram has the same shape as the letter Z, called the Z charge transfer pathway. What is more, it is extensively utilized by researchers to boost the effective separation and migration of charges, facilitate hydrogen generation of water splitting and perform a superior photocatalytic performance. For instance, Xie and co-workers synthesized Ag-AgI/BiOI-Bi_2_O_3_ photocatalyst with Z-scheme multi-charge transfer pathway, which exhibited excellent photocatalytic performance [27]. Qu et al. constructed Ag_2_MoO_4_/Ag/AgBr Z-scheme composites. The obtained composites exhibited an excellent performance for RhB photocatalytic degradation under various reaction conditions [28]. The photocatalysts with Z-scheme photocatalytic charge transfer pathway have different band structures. The p-type transition metal oxide semiconductor, Co_3_O_4_, is a narrow band gap photocatalyst, and has been widely employed as an excellent catalyst [29]. Co_3_O_4_ and g-C_3_N_4_ can meet the requirements of Z-scheme charge transfer pathway due to their suitable valence band edges and conduction band edges [30]. Through the modification of Co_3_O_4_ and construction of Z-scheme charge transfer pathway, an electric field between Co_3_O_4_ and g-C_3_N_4_ photocatalysts is constructed, which is of benefit to the charge separation and migration. Therefore, improved photocatalytic performance can be obtained on Co_3_O_4_/g-C_3_N_4_.

Co_3_O_4_/g-C_3_N_4_ catalysts have been applied to degrade tetracycline [30], several dye pollutants [31] and photocatalytic water oxidation [32]. They all exhibited outstanding photocatalytic performances. For instance, Jin et al. synthesized Co_3_O_4_/g-C_3_N_4_ as peroxymonsulfate-mediated photocatalytic catalysts, which showed good activity for tetracycline degradation [30]. Qu et al. synthesized Co_3_O_4_/g-C_3_N_4_ composites with outstanding photocatalytic peroxymonosulfate activation performance for dyes’ degradation [31].

As has been reported, introducing co-catalysts can greatly improve the hydrogen evolution reaction performance of semiconductors. Researchers have found that modification with noble metal Pt can boost the photocatalysts’ water splitting activity [33,34]. As an electron mediator, Pt has a low Fermi level, which has an important impact on the H_2_ evolution performance.

Based on former reports, g-C_3_N_4_ nanosheets with uniform thickness were synthesized by a polymerization method in the paper. Co_3_O_4_ was then introduced via an electrostatic interaction–calcination method to form Z-scheme charge transfer pathway in order to obtain efficient separation of charge carriers and high photocatalytic water splitting performance. With further Pt modification, better activity was obtained. The Z-scheme photocatalytic charge transfer pathway of Co_3_O_4_/g-C_3_N_4_ was put forward based on the experimental data.

## 2. Materials and Methods

### 2.1. Materials

All the reagents that were used were analytical pure. Triethanolamine (TEOA) was purchased from Aladdin Industry Corporation, Shanghai, China. Melamine was purchased from Tianjin Jiangtian Chemical Technology Co. Ltd., Tianjin, China. Ammonia chloride was purchased from Wind Ship Chemical Reagent Technology Co. Ltd., Tianjin, China. Anhydrous ethanol and ammonium bicarbonate were purchased from Guangfu Technology Development Co. Ltd., Tianjin, China. Cobalt chloride hexahydrate and chloroplatinic acid were purchased from Masco Chemical Co. Ltd., Tianjin, China.

### 2.2. Fabrication of g-C_3_N_4_

The large-scale production of few-layer and ultra-thin g-C_3_N_4_ nanosheets with uniform thickness was performed by the facile method of polymerization. 5 g melamine and 10 g ammonia chloride were fully grinded into powder and then calcinated at 550 °C for 4 h in the air atmosphere. This product was collected and named as g-C_3_N_4_.

### 2.3. Synthesis of the Ultra-Thin Co_3_O_4_/g-C_3_N_4_ Catalysts

The series Co_3_O_4_/g-C_3_N_4_ nanosheets were prepared via electrostatic interaction and calcination methods. 0.72 g g-C_3_N_4_ was sonicated for 0.5 h to disperse in 100 mL anhydrous ethanol. Subsequently, different masses of CoCl_2_·6H_2_O were added into the above system, followed with a stoichiometric ratio of NH_4_HCO_3_, and n(CoCl_2_·6H_2_O): n(NH_4_HCO_3_) = 1:3 [31]. After continuous stirring for 6 h, the sample was centrifugated, washed and dried at 85 ℃. The as-synthesized materials were calcinated at 350 °C for 2 h. The series Co_3_O_4_/g-C_3_N_4_ catalysts with various mass ratios were collected, and are denoted as 0.3, 0.5, 1, 3, 6, 12 wt.% Co_3_O_4_/g-C_3_N_4_, respectively.

For comparison, the pure Co_3_O_4_ was fabricated by calcination of CoCl_2_·6H_2_O at 600 °C for 4 h.

### 2.4. Characterization

The X-ray powder diffraction (XRD) was characterized by a diffractometer from Rigaku Corporation. Scanning electron microscopy image was observed by a SU3500 (Hitachi, Tokyo, Japan) microscope. Elemental analysis was explored by Elemental Analyzer (Vario EL cube, Langenselbold, Germany). The ICP analysis was performed by Inductive Coupled Plasma Emission Spectrometer (SpectroBlue, Kleve, Germany). Transmission electron microscopy (TEM) was observed by a FEI TalosF200X instrument. EDS analyses were characterized by an energy dispersive X-ray spectroscope (Bruker, Billerica, MA, USA). Atomic force microscopy (AFM) images were performed by the Bruker icon AFM instrument. The X-ray photoelectron spectroscopy (XPS, Thermo escalab 250Xi) analysis was characterized on a photoemission spectroscopy, using a mono AlKα radiation source. The UV–Vis diffuse reflection spectra (UV–Vis DRS) was explored by a UV–Vis spectrophotometer (UV3600-Plus, Shimadzu, Kyoto, Japan). Photoluminescence spectra (PL) was conducted by a spectrophotometer (PTI, New York, NY, USA). Electrochemical experiments were explored with a workstation (Zahner Zenium, Kronach, Germany).

### 2.5. Photocatalytic Activity

The H_2_ production tests were implemented with the catalyst (50 mg), which was dispersed in triethanolamine solution (10% vol TEOA) in the Labsolar-6A online system. 0.5 wt.% Pt, which was used as co-catalyst, was in-situ reduced by light on the catalysts by adding H_2_PtCl_6_ aqueous solution in the above suspension of the reaction. After vacuuming, the Argon gas was added. A 300 W Xenon lamp was served to achieve the system irradiation during tests. The solution temperature was controlled at 5 °C to avoid the thermal effect produced by light. Additionally, the tests were carried out using a gas chromatograph and the carrier gas is Argon (GC D7900 II, Tianmei, Shanghai, China).

## 3. Results and Discussion

### 3.1. XRD Analysis

**Figure 1:**
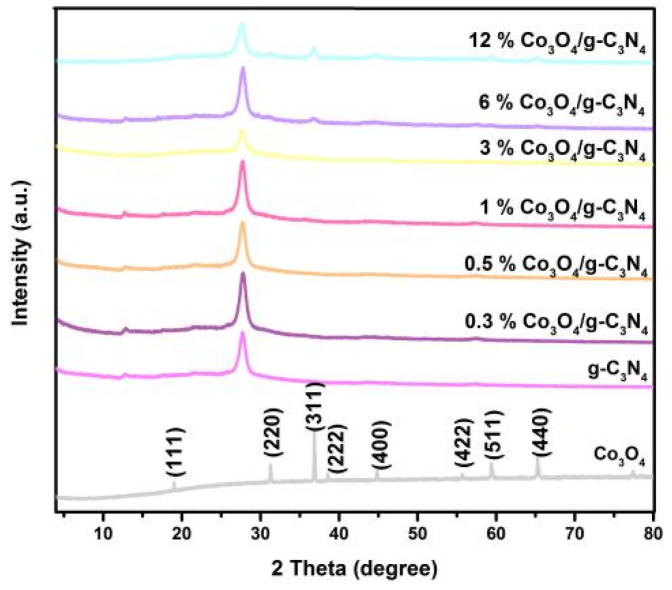
XRD pattern of fabricated samples.

The XRD measurement was characterized to confirm the phase of the catalysts, as shown in Figure 1. The peaks at 2θ = 13. 0 and 27.7° were indexed to the characteristic peaks of g-C_3_N_4_ [5,35]. The diffraction peaks at 2θ = 19.0°, 31.3°, 36.9°, 38.5°, 44.8°, 55.7°, 59.4° and 65.2° were indexed as (1 1 1), (2 2 0), (3 1 1), (2 2 2), (4 0 0), (4 2 2), (5 1 1) and (4 4 0) plane of cubic Co_3_O_4_ (PDF#42-1467), respectively. It was evident that the peaks of Co_3_O_4_ and g-C_3_N_4_ were both existed in the 6% and 12% Co_3_O_4_/g-C_3_N_4_ photocatalysts’ XRD patterns. No obvious Co_3_O_4_ peaks were observed in Co_3_O_4_/g-C_3_N_4_ samples with 0.3%, 0.5%, 1% and 3% Co_3_O_4_. That might be owing to the relatively low content and high distribution of Co_3_O_4_ [36]. What is more, as clearly demonstrated, the peaks of the photocatalysts were sharp and strong, illustrating that the samples possessed an excellent crystallization. There were no peaks of other phases in Figure 1, which indicated the good purity of the synthesized photocatalysts.

### 3.2. EA and ICP

For the g-C_3_N_4_ sample, the atomic ratio of C element and N element was investigated by EA. The value exhibited a result that the atomic ratio of C/N = 3.00: 4.27, which was very close to the value of g-C_3_N_4_. Based on XRD analysis results, the as-synthesized products were pure g-C_3_N_4_.

The amounts of Co element in the as-prepared catalysts were monitored by the ICP. The actual contents of 0.3%, 0.5%, 1%, 3%, 6% and 12% Co_3_O_4_/g-C_3_N_4_ is 0.3540, 0.5354, 1.146, 3.038, 6.253, 12.44 wt.%, respectively. The corresponding contents of Co_3_O_4_ in the series Co_3_O_4_/g-C_3_N_4_ photocatalysts are close to theoretical values. Due to the negative zeta potentials of g-C_3_N_4_, there was strong electrostatic interaction between Co^2+^ and g-C_3_N_4_ nanosheets [37]. As a result, the added Co^2+^ should have no considerable loss. Actually, the actual weight ratio of Co_3_O_4_ to g-C_3_N_4_ in the prepared Co_3_O_4_/g-C_3_N_4_ catalysts was a little higher than original CoCl_2_·6H_2_O to g-C_3_N_4_. It was assignable to the slight weight loss after the second calcination process of g-C_3_N_4_.

### 3.3. Morphology Analysis

**Figure 2:**
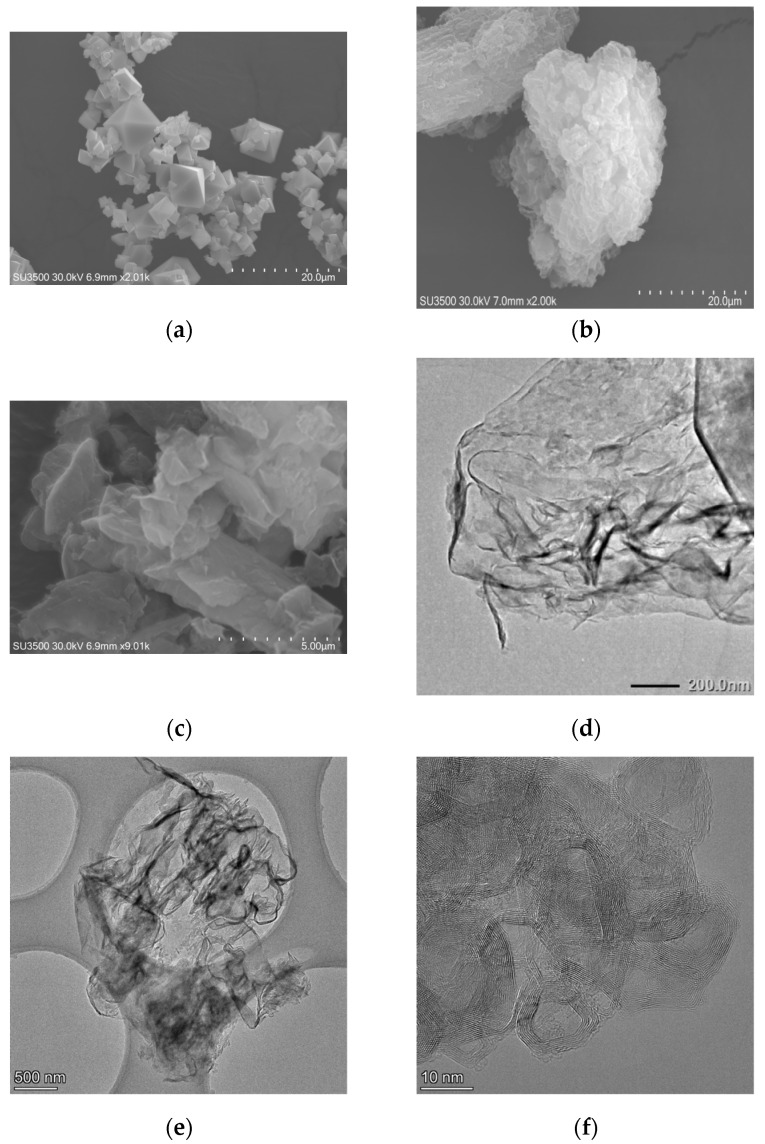
SEM images of (**a**) Co_3_O_4_, (**b**) 0.5% Co_3_O_4_/g-C_3_N_4_, and (**c**) Pt/0.5% Co_3_O_4_/g-C_3_N_4_. The TEM image of (**d**) 0.5% Co_3_O_4_/g-C_3_N_4_ and (**e**,**f**) Pt/0.5% Co_3_O_4_/g-C_3_N_4_.

**Figure 3:**
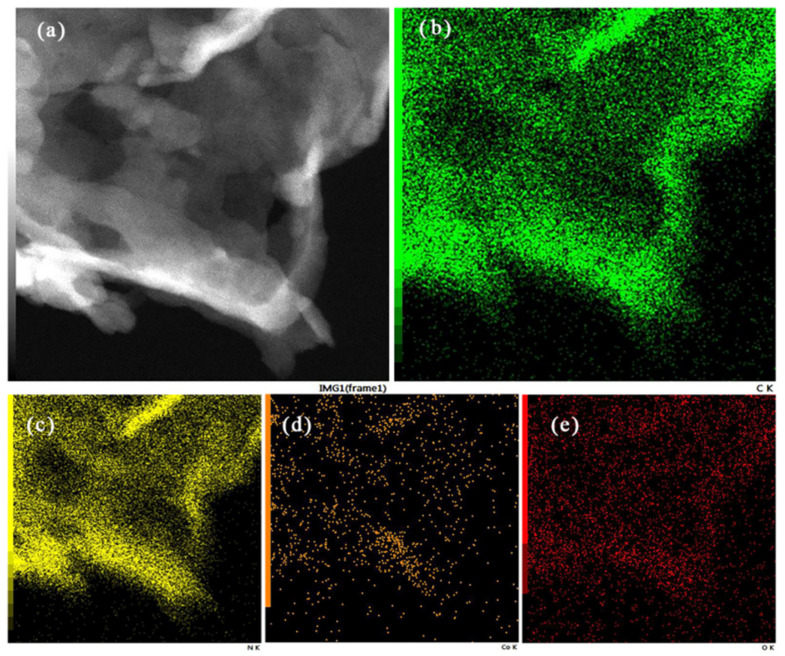
(**a**) The TEM image of 0.5% Co_3_O_4_/g-C_3_N_4_. (**b**–**e**) Elemental maps of the catalyst: (**b**) C, (**c**) N, (**d**) Co and O elements.

**Figure 4:**
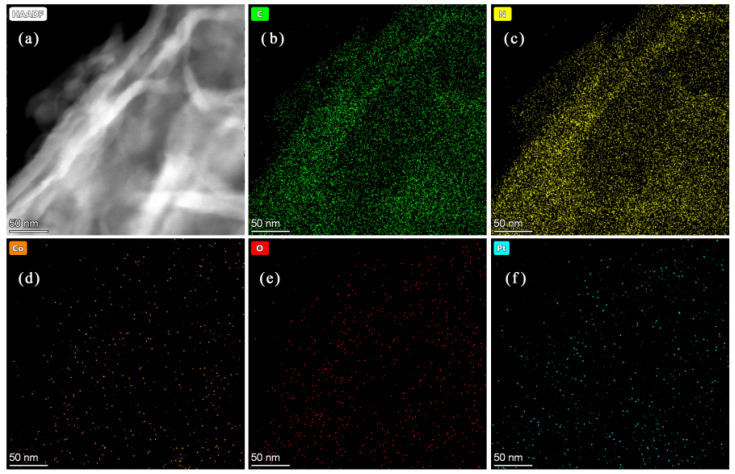
(**a**) HAADF-TEM image of Pt/0.5% Co_3_O_4_/g-C_3_N_4_. (**b**–**f**) Elemental maps of the catalyst: (**b**) C, (**c**) N, (**d**) Co, (**e**) O and (**f**) Pt elements.

The microstructure of as-fabricated catalysts was presented in Figure 2. From Figure 2a, Co_3_O_4_ had polyhedral structure, and the 0.5% Co_3_O_4_/g-C_3_N_4_ showed an irregular and crinkly structure. Pt/0.5% Co_3_O_4_/g-C_3_N_4_ exhibited 2D morphology with a crinkly nanosheet structure, which seem to be a loose and soft product with a diameter of several micrometers. As observed in the images, the microstructures of 0.5% Co_3_O_4_/g-C_3_N_4_ and Pt/0.5% Co_3_O_4_/g-C_3_N_4_ were nanosheets. The composition and elements distribution of 0.5% Co_3_O_4_/g-C_3_N_4_ and Pt/0.5% Co_3_O_4_/g-C_3_N_4_ were further investigated by EDS maps, as displayed in Figure 3 and Figure 4. All of the elements were uniformly dispersed in the photcatalysts, and no signals of other elements were observed. Furthermore, the existence of C, N, Co, O and Pt were highly coincided with the area of g-C_3_N_4_. It seems that Co_3_O_4_ and Pt could be uniformly deposited on the g-C_3_N_4_.

### 3.4. AFM Analysis

**Figure 5:**
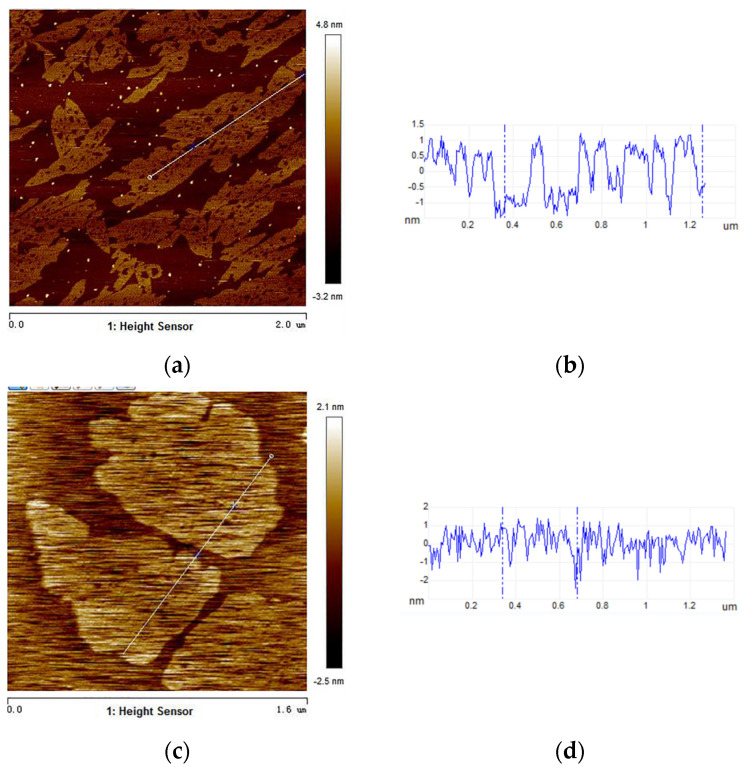
(**a**,**b**) AFM images and corresponding thickness of pure 2D g-C_3_N_4_ and (**c**,**d**) 0.5 % Co_3_O_4_/g-C_3_N_4_.

To further investigate the structure and height profiles of the photocatalysts, AFM was performed. The images and corresponding thickness of samples were presented in Figure 5. The architectures of the g-C_3_N_4_ were 2D ultra-thin nanosheets, with 1–2 nm step height. As illustrated in Figure 5c,d, there is no obvious step height change for 0.5% Co_3_O_4_/g-C_3_N_4_ ultra-thin nanosheets. Additionally, g-C_3_N_4_ and 0.5% Co_3_O_4_/g-C_3_N_4_ nanosheets both exhibited few-layer ultra-thin nanosheets with uniform thickness. Ultrathin nanosheets had a short bulk diffusion length [22,38]. Compared with bulk g-C_3_N_4_, ultrathin g-C_3_N_4_ nanosheets and 0.5% Co_3_O_4_/g-C_3_N_4_ photocatalysts had a shorter electron transfer path, which can increase the lifetime of photoexcited electrons. The unique structure of few-layer g-C_3_N_4_ photocatalyst generated numerous charge transfer nanochannels and provided a short transfer path for electron transfer, which would promote the efficient separation and migration of e^−^ and h^+^ [22].

### 3.5. XPS Analysis

**Figure 6:**
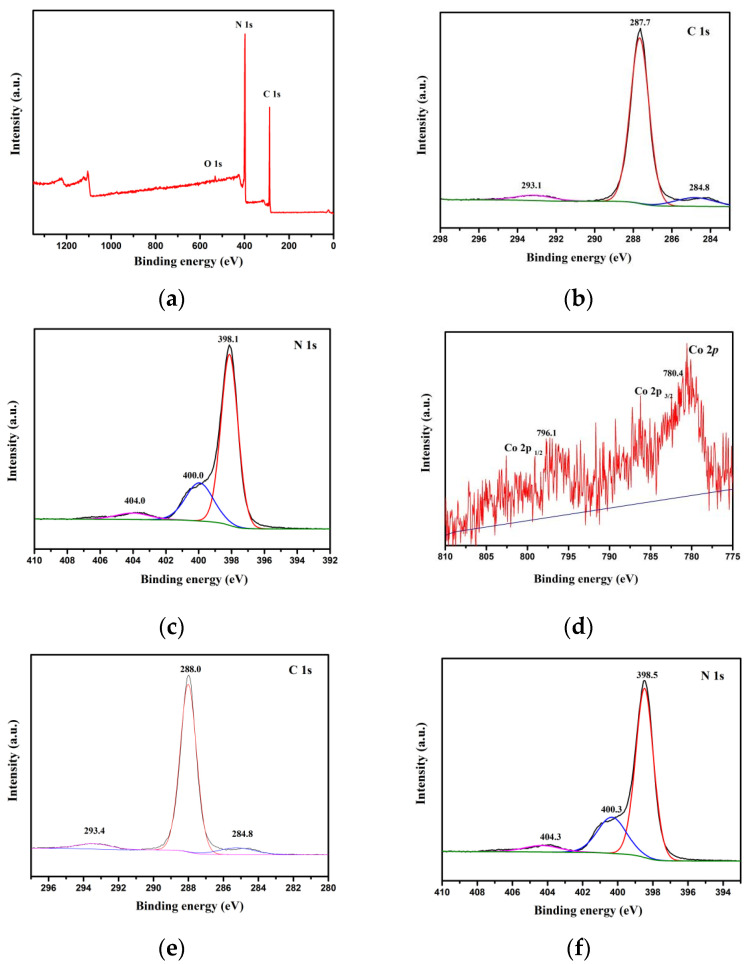
XPS spectra (**a**–**d**) survey, C 1s, N 1s and Co 2p of 0.5 % Co_3_O_4_/g-C_3_N_4_, (**e**,**f**) C 1s and N 1s of g-C_3_N_4_, respectively.

To confirm the element compositions and chemical states in the as-synthesized 0.5% Co_3_O_4_/g-C_3_N_4_, the XPS analysis was conducted. From XPS survey spectrum (Figure 6a), C 1s, N 1s and O 1s peaks were evident. What is more, no signals assigned to other elements were displayed in this survey spectrum, suggesting that the purity of the samples is very high. As displayed in Figure 6b, the peak at 287.7 eV is due to sp^2^ C atoms, which can bond to N atoms in catalysts [39]. Additionally, the peak at 293.1 eV is owing to π-excitation [40]. Evidently, N 1s has three different peaks at 398.1 eV, 400.0 eV and 404.0 eV in Figure 5c assigning to sp^2^ N atoms of triazine rings, bridging nitrogen atoms in (N–(C)_3_) and the charging effects of the hetero-cycles or positive charge localization [41], respectively. The Co 2p at the peaks of 780.4 and 796.1 eV are ascribed to Co 2p_3__/2_ and Co 2p_1__/2_, suggesting that Co_3_O_4_ exists in the photocatalysts [42,43]. Compared with g-C_3_N_4_, there was negative energy shift of C 1s and N 1s in 0.5% Co_3_O_4_/g-C_3_N_4_ photocatalysts, indicating that the e^−^ are transferred from Co_3_O_4_ to g-C_3_N_4_ [31]. As discussed in the Z-scheme charge transfer system, e^−^ on CB of Co_3_O_4_ can migrate to VB of the other semiconductor. Therefore, Z-scheme charge transfer pathway of Co_3_O_4_/g-C_3_N_4_ system agreed with the results of negative binding energy shift in 0.5% Co_3_O_4_/g-C_3_N_4_ photocatalysts. These results take firm evidence that the Co_3_O_4_ and g-C_3_N_4_ were co-existed in the samples and Z-scheme charge transfer pathway was constructed in photocatalysts.

### 3.6. UV–Vis DRS Analysis

**Figure 7:**
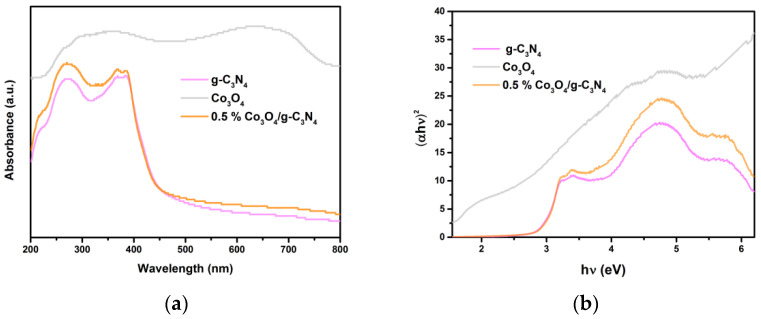
(**a**) UV–Vis DRS of the catalysts. (**b**) Bandgap energies of the photocatalysts.

The UV–Vis DRS spectrophotometer analysis was performed to measure optical absorption properties of the catalysts. 0.5% Co_3_O_4_/g-C_3_N_4_ possessed a strong absorption and its edge located at 443 nm in Figure 6a. Additionally, although the absorption edges and bandgap energies of g-C_3_N_4_ and 0.5% Co_3_O_4_/g-C_3_N_4_ had no apparent distinction, optical absorption intensity of 0.5% Co_3_O_4_/g-C_3_N_4_ was apparently increased, which made it easier to harvest light. The spectra (Figure 7a) of the catalysts suggested that the samples displayed a strong absorption all between 200–440 nm, which covered ultraviolet and visible regions. These photocatalysts can respond to the visible light and their responsiveness was enhanced, which has probably a more important advantage to photocatalytic water splitting. Bandgaps of Co_3_O_4,_ g-C_3_N_4_ and 0.5% Co_3_O_4_/g-C_3_N_4_ photocatalysts were achieved by the Tauc plots, and results are presented in Figure 7b. The bandgap (E_g_) of catalysts can be received by the Tauc Equation [44]. Because the Co_3_O_4_ [45] and g-C_3_N_4_ [46] are typical direct semiconductors, n in the Tauc equation should be all 1/2. The E_g_ of as-prepared g-C_3_N_4_ and Co_3_O_4_ are 2.8 eV and 2.0 eV, respectively. The E_CB_ values of Co_3_O_4_ and g-C_3_N_4_ are obtained via this equation.

(1) $$E_{\mathrm{CB}}{=\chi \;-\;E}_{e}\;-\;0{.5E}_{g}$$

χ for g-C_3_N_4_ and Co_3_O_4_ are 4.64 [47] and 5.90 [48], respectively. E_CB_ of g-C_3_N_4_ and Co_3_O_4_ are −1.26 eV and 0.4 eV, respectively. So E_VB_ values of g-C_3_N_4_ and Co_3_O_4_ are 1.54 eV and 2.4 eV, respectively. Because of the appropriate band structure of two semiconductors, the e^−^ would like to transfer from CB of Co_3_O_4_ to VB of g-C_3_N_4_. Finally, e^−^ and h^+^ accumulate at CB of g-C_3_N_4_ and VB of Co_3_O_4_, respectively. Charge carriers can be effectively separated. These data of g-C_3_N_4_ and Co_3_O_4_ can be known based on the formulas and experiments.

### 3.7. PL

**Figure 8:**
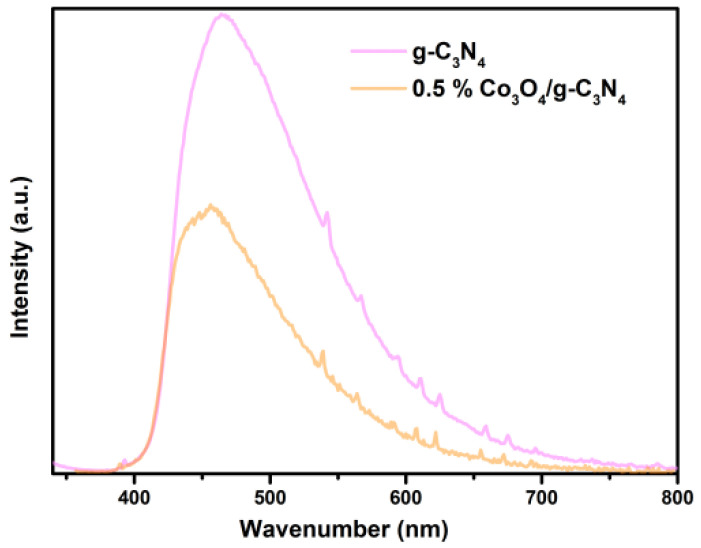
Steady-state photoluminescence spectra of fabricated photocatalysts.

As displayed in Figure 8, the recombination ability of e^−^ and h^+^ was speculated from the photoluminescence intensity of the samples. All the photocatalysts were evaluated at the excitation wavelength, which was 320 nm. Generally, the higher intensity of the PL emission spectra means a worse separation ability of carriers [49]. The spectrum of 0.5% Co_3_O_4_/g-C_3_N_4_ catalyst showed lower emission intensity than the intensity of g-C_3_N_4_, leading to a lower recombining frequency of e^−^ and h^+^ in 0.5% Co_3_O_4_/g-C_3_N_4_. The lower the peak of photoluminescence spectra presented, the more excellent catalytic performance can be anticipated. It seems that 0.5% Co_3_O_4_/g-C_3_N_4_ catalyst may have a better photocatalytic performance than pure g-C_3_N_4_.

### 3.8. Photoelectrochemical Analysis

**Figure 9:**
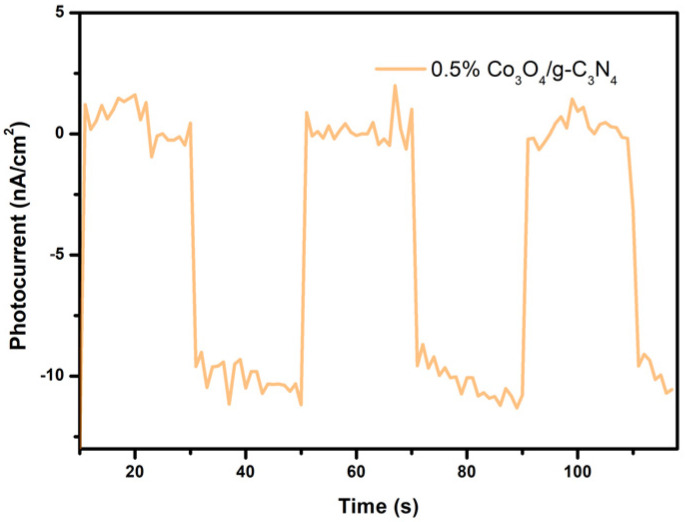
The photocurrent measurement spectra of the 0.5% Co_3_O_4_/g-C_3_N_4_ catalysts.

The photocurrent measurement was produced under the irradiation of 429 nm wavelength light, as illustrated in Figure 9. The photocurrent immediately increased to a value and remained when the light irradiated on the catalysts. The photocurrent decreased as soon as the light turned off. It manifested that 0.5% Co_3_O_4_/g-C_3_N_4_ has a photocurrent response and the photocurrent value is around 10 nA at 0.1 V. However, no photocurrent existed in g-C_3_N_4_. It indicated that 0.5% Co_3_O_4_/g-C_3_N_4_ is visible to light-responsive photocatalysts. After Co_3_O_4_ was introduced, the g-C_3_N_4_ catalyst can respond to visible light, and a photocurrent was exhibited. That should be attributed to the formation of Z-scheme charge transfer pathway, which resulted in improved separation and migration of the photo-induced charges. As a result, a photocurrent with higher intensity can be obtained.

### 3.9. Photocatalytic Activity and Cycling Tests

**Figure 10:**
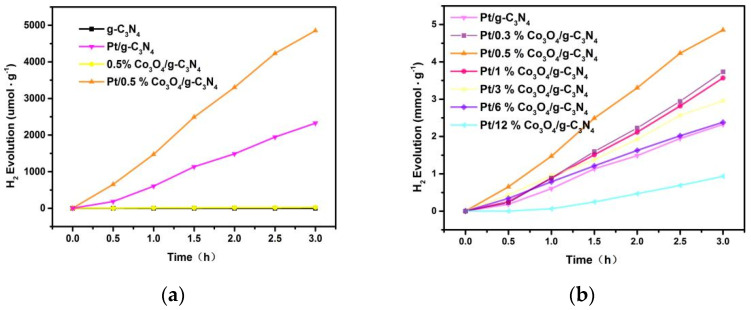
(**a**,**b**) Photocatalytic hydrogen evolution performance of prepared catalysts.

As illustrated in Figure 10a, g-C_3_N_4_ exhibits no activity. However, the series Co_3_O_4_/g-C_3_N_4_ catalysts all present good photocatalytic performance, and 0.5% Co_3_O_4_/g-C_3_N_4_ has the fine H_2_ evolution rate of 8.774 μmol∙g^−1^∙h^−1^. Obviously, the modification of Co_3_O_4_ can greatly promote the water splitting reaction rate of g-C_3_N_4_. The results of photocatalytic activity tests can be proved by photoelectrochemical analysis. As indicated in the photoelectrochemical analysis, the g-C_3_N_4_ can respond to 429 nm only after Co_3_O_4_ was modified. Therefore, the quantum yield of g-C_3_N_4_ is low. Catalytic activity of g-C_3_N_4_ is also low. The photoluminescence spectra analysis showed that e^−^ and h^+^ in the g-C_3_N_4_ have higher recombination rates. It suggested that g-C_3_N_4_ has a lower photocatalytic hydrogen production.

**Figure 11:**
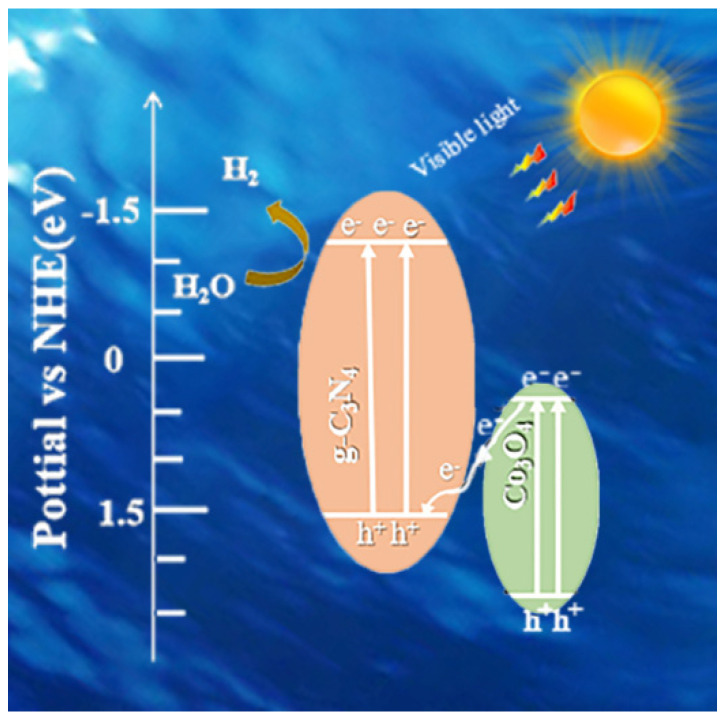
Proposed mechanism of photocatalytic system.

According to the band structure of Co_3_O_4_ and g-C_3_N_4_ as we noted above, the reasonable photocatalytic charge transfer pathway was preliminarily put forward to explain the reason for catalytic improved-performance of 0.5% Co_3_O_4_/g-C_3_N_4_ in Figure 11. The electrons of Co_3_O_4_ and g-C_3_N_4_ are photo-generated from VB to CB in response to irradiation. After calcination, interaction between Co_3_O_4_ and g-C_3_N_4_ was enhanced. The e^−^ on CB of Co_3_O_4_ would quickly migrate to VB of g-C_3_N_4_ and combine with h^+^, which replaced inner self-combination. Some holes were reacted with TEOA, which worked as an oxidation sacrificial agent. Therefore, the electric field between Co_3_O_4_ and g-C_3_N_4_ was formed, with electrons and holes accelerated at different parts. The electric field can promote the directional transfer of charges and introduction of Co_3_O_4_ can boost the electric field of Co_3_O_4_/g-C_3_N_4_. Hence, the existence of Co_3_O_4_ effectively promoted the interfacial charges to separate and transfer and the hydrogen generation, leading to superior photocatalytic performance. What is more, the XPS analysis indicated that e^−^ are transferred from Co_3_O_4_ to g-C_3_N_4_ nanosheets. It suggested that the Z-scheme charge transfer pathway was in agreement with experiment results.

The probable reactions process of photocatalytic hydrogen generation is as follows:

(2) $${\mathrm{Co}}_{3}O_{4}{/g\text{-}C}_{3}N_{4}+\mathrm{hv}\;\to {\;\mathrm{Co}}_{3}O_{4}{(e}^{-}{+h}^{+}{)/g\text{-}C}_{3}N_{4}{(e}^{-}{+h}^{+})$$

(3) $$h_{\mathrm{VB}}^{+}{(\mathrm{Co}}_{3}O_{4})+\mathrm{TEOA}\;\to {\;\mathrm{oxidation}\;\mathrm{product}\;\mathrm{of}\;\mathrm{sacrificial}\;\mathrm{reagent}}_{\;}$$

(4) $$e_{\mathrm{CB}}^{-}{(g\text{-}C}_{3}N_{4}{)+2H}_{2}O\;\to {\;H}_{2}{+2\mathrm{OH}}^{-}$$

Thence, the proposed Z-scheme charge transfer pathway of photocatalysts showed good separation and migration of e^−^ and h^+^. The obvious enhancement of the hydrogen generation ability of the 0.5% Co_3_O_4_/g-C_3_N_4_ ultra-thin nanosheets may attribute to the significant and ideal Z-scheme photocatalytic charge transfer pathway.

After Pt was added, further efforts were made to improve the performance of Co_3_O_4_/g-C_3_N_4_. As presented in Figure 10a, Pt/0.5% Co_3_O_4_/g-C_3_N_4_ exhibited a predominant rate and the average rate was up to 1620 μmol∙g^−1^∙h^−1^. This is 2.1 times the photocatalytic performance of Pt/g-C_3_N_4_.

To explore the influence of Co_3_O_4_ contents on the performance of the Pt/Co_3_O_4_/g-C_3_N_4_, results of Pt/Co_3_O_4_/g-C_3_N_4_ with different Co_3_O_4_ contents were shown in Figure 10b. From this figure, it can be observed that all photocatalysts manifested improved hydrogen generation activity compared with Pt/g-C_3_N_4_, except Pt/12% Co_3_O_4_/g-C_3_N_4_. Owning to the relatively low content of Co_3_O_4_ (0.5 % Co_3_O_4_), Pt was mainly deposited on g-C_3_N_4_ nanosheets. When the catalyst was illuminated, the electrons migrated from Co_3_O_4_ to g-C_3_N_4_, and were finally transferred to Pt. The H^+^ in the solution obtained the electrons from the Pt particles to form H_2_. When the Co_3_O_4_ content was relatively low (0.3%), Z-scheme charge transfer pathway is still the main pathway in this photocatalytic system. However, the amount of electrons and holes in Co_3_O_4_ was relatively low than that in the 0.5% one. Therefore relatively large amounts of holes in g-C_3_N_4_ were reacted with TEOA instead of combining with the electrons in Co_3_O_4_. As a result, e^−^ and h^+^ in the Pt/0.3% Co_3_O_4_/g-C_3_N_4_ have higher recombination rates than in Pt/0.5% Co_3_O_4_/g-C_3_N_4_, and the Pt/0.3% Co_3_O_4_/g-C_3_N_4_ exhibited lower photoactivity. When Co_3_O_4_ contents increased, Co_3_O_4_ will accumulate. More and more Pt/Co_3_O_4_ interfaces formed, and the electrons migrated from Co_3_O_4_ to Pt, instead of combining with the holes in g-C_3_N_4_. Consequently, the Z-scheme charge transfer pathway was blocked, and the activity of Co_3_O_4_/g-C_3_N_4_ decreased with the increasing Co_3_O_4_ contents.

**Figure 12:**
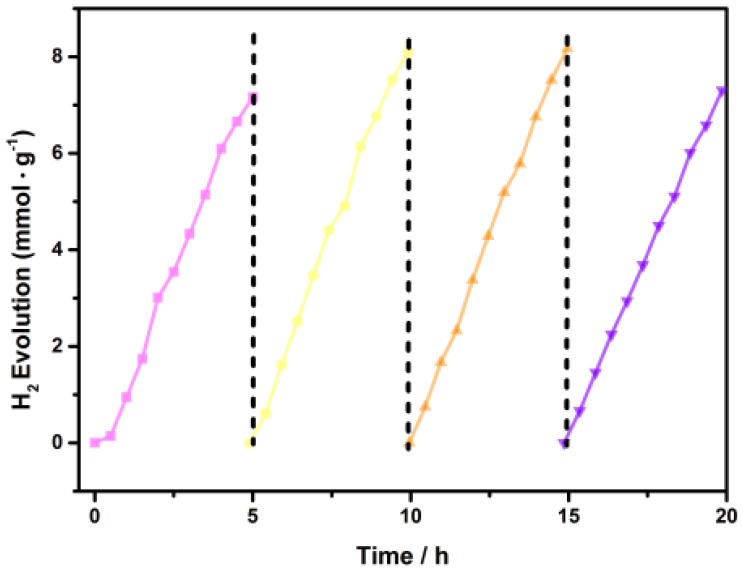
Photocatalytic stability performance of Pt/0.5% Co_3_O_4_/g-C_3_N_4_.

Cycling stability tests of the optimal Pt/0.5% Co_3_O_4_/g-C_3_N_4_ were evaluated by a recycle experiment, which was presented in Figure 12. It could suggest that after four successive cycles, no noticeable diminution was observed within 20 h with TEOA as sacrificing agents, suggesting that the Pt/0.5% Co_3_O_4_/g-C_3_N_4_ photocatalyst had an excellent stability and it is reusable for practical applications.

## 4. Conclusions

In this paper, g-C_3_N_4_ ultra-thin nanosheets were fabricated via the polymerization method. The Co_3_O_4_/g-C_3_N_4_ series were fabricated by an electrostatic interaction-calcination treatment. The various characterization results indicated that the Co_3_O_4_/g-C_3_N_4_ catalysts exhibit Z-scheme charge transfer pathway, and possessed excellent photocatalytic hydrogen evolution performance with high stability and fine charge transfer ability. After Pt is added, the Co_3_O_4_/g-C_3_N_4_ exhibited better activity, and Pt/0.5% Co_3_O_4_/g-C_3_N_4_ exhibited the best activity, with a hydrogen generation rate of 1620 μmol∙g^−1^∙h^−1^. Based on the characterization and environment data, the catalytic reaction charge transfer pathway of Pt/Co_3_O_4_/g-C_3_N_4_ was proposed. Therefore, the work provided a promising method to design efficient and reusable photocatalytic systems with Z-scheme charge transfer pathways for solar-energy conversion. Further practical applications of this Z-scheme charge transfer pathway were anticipated.

## Data Availability

The data presented in this study are available on request from the corresponding author.
